# Consolidative Hematopoietic Stem Cell Transplantation After CD19 CAR-T Cell Therapy for Acute Lymphoblastic Leukemia: A Systematic Review and Meta-analysis

**DOI:** 10.3389/fonc.2021.651944

**Published:** 2021-04-28

**Authors:** Xinjie Xu, Sifei Chen, Zijing Zhao, Xinyi Xiao, Shengkang Huang, Zhaochang Huo, Yuhua Li, Sanfang Tu

**Affiliations:** ^1^ The Second School of Clinical Medicine, Zhujiang Hospital, Southern Medical University, Guangzhou, China; ^2^ Department of Hematology, Zhujiang Hospital, Southern Medical University, Guangzhou, China; ^3^ State Key Laboratory of Cardiovascular Disease, Fuwai Hospital, National Center for Cardiovascular Diseases, Chinese Academy of Medical Sciences and Peking Union Medical College, Beijing, China

**Keywords:** meta-analysis, acute lymphoblastic leukemia, CD19, hematopoietic stem-cell transplantation, chimeric antigen receptor T (CAR-T)

## Abstract

**Background:**

This study aimed to systematically evaluate and compare the efficacy and safety of consolidative hematopoietic stem cell transplantation (HSCT) after CD19 chimeric antigen receptor T (CAR-T) therapy with non-HSCT in the treatment of acute lymphoblastic leukemia (ALL).

**Methods:**

The PubMed, Embase, Cochrane Library and Web of Science databases were searched for clinical trials. Pooled hazard ratios (HRs) for overall survival (OS), relapse rate, and leukemia-free survival (LFS) as well as overall incidence rates for transplant-related mortality (TRM), acute graft-*versus*-host disease (aGVHD), chronic graft-*versus*-host disease (cGVHD), and infections were calculated using Stata software.

**Results:**

We screened 3,441 studies and identified 19 eligible studies with 690 patients. Among the patients who achieved complete remission (CR) after CD19 CAR-T therapy, consolidative HSCT was beneficial for OS (HR = 0.34, 95% CI, 0.170.68, P = 0.003), the relapse rate (HR = 0.16, 95% CI, 0.100.25, P < 0.001), and LFS (HR = 0.15, 95% CI, 0.080.28, P < 0.001). For patients who achieved MRD-negative (neg) CR after CD19 CAR-T therapy, consolidative HSCT was beneficial for OS (0.57, 95% CI, 0.330.99, P = 0.045), the relapse rate (0.14, 95% CI, 0.060.31, P < 0.001), and LFS (0.21, 95% CI, 0.120.35, P < 0.001). Regarding safety, we calculated pooled incidence rates for TRM (8%, 95% CI, 0.020.15), aGVHD (44%, 95% CI, 0.230.67), cGVHD (36%, 95% CI, 0.170.56), and infections (39%, 95% CI, 0.030.83).

**Conclusions:**

Compared with non-HSCT treatment, consolidative HSCT after CD19 CAR-T therapy for R/R B-ALL patients can prolong OS and LFS and reduce the risk of relapse. The incidence rates for adverse events are acceptable. More high-quality randomized controlled trials are required to avoid bias and further determine the efficacy of HSCT.

## Introduction

B-cell acute lymphoblastic leukemia (B-ALL) is one of the most common hematological malignancies in children and young adults. Patients with refractory or relapsed (R/R) B-ALL are usually resistant to traditional therapy and have a poor prognosis. Novel therapeutic strategies to improve prognosis have been widely studied, and chimeric antigen receptor (CAR)-T cell therapy is a promising and potent approach. T cells with engineered CAR molecules recognize antigens on tumor cells *via* single-chain variable fragments (scFv) without MHC restriction. High complete remission (CR) rates were reported in different clinical trials of CD19 CAR-T therapy; in 2017, the first CD19 CAR-T producttisagenlecleucelwas approved by the Food and Drug Administration (FDA).

However, relapse is a challenge for CAR-T therapy, with rates ranging from 20 to 70% during sufficiently long follow-up periods (1). A combinatorial treatment modality might be needed to enhance the antitumor effect. Allogeneic hematopoietic stem cell transplantation (allo-HSCT), the only potentially curative therapy for R/R B-ALL (2, 3), is considered a promising option. CAR-T cells may be used as a bridge to HSCT because they can induce a high CR rate. HSCT rebuilds the patients hematopoietic and immune system. Genetic differences between patients and donors will induce graft *versus* leukemia (GVL) effects, thus maintaining the patients long-term remission and reducing the relapse rate. However, whether to administer HSCT after CAR-T therapy remains controversial. Because CAR-T cells can survive and maintain their therapeutic effect *in vivo* for a long time, patients may have similar outcomes regardless of whether they undergo subsequent consolidative HSCT. Furthermore, transplant-associated complications, particularly lethal complications, may severely affect the patient prognosis. Thus, whether bridging HSCT benefits ALL patients remains unclear.

Recently, an increasing number of clinical trials have compared the outcomes of patients with and without transplantation after CD19 CAR-T therapy. This study aimed to evaluate the efficacy and safety of HSCT after CAR-T therapy to treat R/R B-ALL and provide more objective data for optimal clinical practices.

## Methods

This study was conducted in accordance with the Preferred Reporting Items for Systematic Reviews and Meta-analyses (PRISMA) reporting guidelines (4) and was registered in PROSPERO on July 5, 2020. The registration number is CRD42020182281.

### Data Sources and Searches

We searched the PubMed, Embase, Cochrane Library and Web of Science databases using the key terms receptor, chimeric antigen, hematologic malignancy, and hematopoietic stem cell transplantation. All relevant studies published from 01 January 2011 to 03 August 2020 were searched without restriction on country or article type. The reference lists of all the selected articles were independently screened to identify additional studies.

### Study Selection

The inclusion criteria were as follows: (1) clinical trials; (2) patients with a diagnosis of relapsed/refractory B-lineage ALL without other hematological malignancies; (3) patients receiving CD19 CAR-T therapy followed by consolidative HSCT; (4) reported necessary information, such as clear grouping of patients and follow-up data; (5) studies conducted from 2011 to the present. Unpublished gray literature, commentaries, letters, reviews, and editorials were excluded. Studies with the same clinical trial number were excluded or analyzed for different outcomes.

### Data Extraction

The data were independently extracted by two reviewers (SC and ZZ) using a standardized collection form. XXu confirmed the data and adjudicated any remaining discrepancies. The hazard ratios (HRs) of six trials (5,10) selected for the efficacy analysis were not reported; thus, they were extracted based on data in waterfall plots using WebPlotDigitizer (version 4.2). The relapse rate and survival status were converted into HR by SPSS (version 25). The data from one trial (11) presented in the Kaplan-Meier curve were extracted using the automatic point-finding method of the Engauge Digitizer (version 11.1) and Photoshop (version 2020), and then the data were entered into an HR calculation spreadsheet template developed by Sydes and Tierney (12). The estimated HR and 95% confidence interval (CI), under the assumption of uniform within-interval censoring, were used in the analysis. Data concerning transplant-related adverse events were extracted in the text, and the total number of target events in the HSCT group were collected.

### Data Analysis

All statistical analyses were conducted using Stata version 16.0 and Stata version 14.0 (StataCorp, College Station, TX, USA). The pooled HR was used to describe the outcome indicators of OS, relapse and LFS. The command metaprop (13) was used in Stata to analyze the rates of transplant-related adverse events. The 95% CI was selected, and P < 0.05 indicated statistically significant results. We used Cochrans Q test and I^2^ statistic to assess heterogeneity between studies. In the former, P < 0.1 indicated significant heterogeneity. If I^2^ < 50%, the fixed-effects model was selected; if I^2^ 50%, the random-effects model was chosen due to substantial heterogeneity. Sensitivity analysis was performed to evaluate the stability of the results.

The Newcastle-Ottawa scale was selected to evaluate cohort study quality. The modified Institute of Health Economics (IHE) tool was adopted to evaluate the study quality of single-arm studies (14). We assessed publication bias using funnel plots, Beggs test, and Eggers test and defined significant publication bias as a P value <0.05.

## Results

### Study Selection

**Figure 1:**
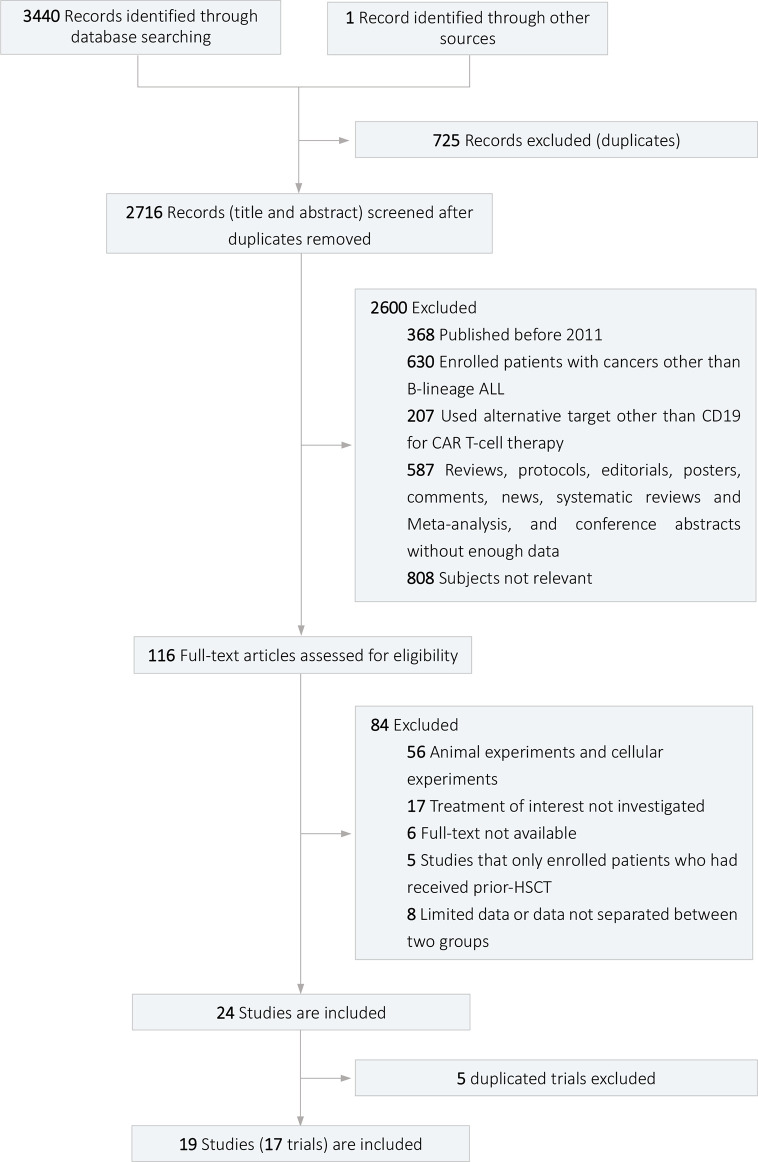
Flow diagram of the study selection process.

The flow diagram of the study selection process is shown in **Figure 1**. In total, 3,441 articles were retrieved from four databases and other sources (668 from PubMed, 1,375 from Web of Science, 1,134 from Embase, 263 from Cochrane Library, 1 from other sources), of which 19 (1, 5,11, 15,25) were included for quantitative analysis. The 19 studies were from 17 different clinical trials, and studies from the same trials were analyzed for different outcomes. Of the 19 studies with 690 patients, 11 reported data related to efficacy outcomes, and 14 reported data concerning safety outcomes. Eleven (1, 5,11, 16, 19, 24) studies were cohort studies, and 8 (15, 17, 18, 20,23, 25) were single-arm studies.

### Study Characteristics

All 11 studies enrolled for efficacy analysis were cohort studies. The basic information of the 11 enrolled studies is shown in **Table 1**; 586 R/R ALL patients received CD19 CAR-T infusion. Additionally, 521 patients achieved CR/CRi, and 456 achieved MRD-negative (neg) CR. Regarding the costimulatory domain of CAR-T cells, three studies used CD28, seven used 4-1BB, and one adopted both CD28 and 4-1BB. A total of 266 patients were bridged to HSCT after achieving CR, and 218 were bridged to HSCT after achieving MRD-neg CR. The median time between CAR-T infusion and HSCT was within 3 months for the eight studies that reported the median interval time. Ten of the 11 studies performed allogeneic transplantation, and one (16) performed allogeneic or autologous transplantation.

**Table 1:** Characteristics of the included studies with efficacy outcomes.

Study	Location	Number of patients	Median Age (range)	Number of patients with CR (%)	MRD-negative CR (method)	Number of CR patients receiving consolidative HSCT	Number of MRD-neg CR patients receiving consolidative HSCT	Chemotherapy(N)	CAR costimulatory domain	Median days post-CAR-T to transplantation (range)	Toxicity (N)
Park (2018) **(** 11 **)**	USA	53	44 (2374)	44 (83.0)	32 (FCM)	17	16	C y(43) Cy/Flu (10)	CD28	74 (44312)	CRS (14) NT (23) Multiorgan failure (1)
Lee (2016) **(** 19 **)**	USA	51	NR	31 (60.8)	28 (NR)	21	21	LDflu/Cy (35) FLAG (6) ifosfamide/Vp16 (2) HDflu/Cy (8)	CD28	54 (NR)	CRS (7) NT (3) Seizures (2)
Jacoby (2018) **(** 7 **)**	Israel	20	11 (548)	18 (90.0)	11 (PCR)	14	NR	Cy/Flu	CD28	68 (NR)	CRS (16) NT (11)
Turtle (2016) **(** 9 **)**	USA	30	40 (2073)	29 (96.7)	27 (FCM/qPCR/karyotyping/FISH)	13	13	Cy/Flu (17) Cy (11) CE (2)	4-1BB	NR	CRS (25) NT (15) BCA (29)
Gardner (2017) **(** 5 **)**	USA	43	12.2 (1.325.4)	40 (93.0)	40 (FCM)	11	11	Cy/Flu (14) Cy (27)	4-1BB	NR	CRS (40) NT (21) BCA (40)
Cao (2018) **(** 16 **)**	China	18	14 (357)	14 (77.8)	12 (FCM)	5	5	Cy/Flu	4-1BB	65 (30138)	CRS (17) NT (1) BCA (14)
Jiang (2019) **(** 8 **)**	China	58	NR	51 (87.9)	47 (FCM)	21	21	Vp16/Bu/Cy	4-1BB	Within 90	CRS (22) NT (9)
Gu (2020) **(** 6 **)**	China	56	All: 34 (1859) HSCT: 41 (1659)	51 (91.1)	38 (FCM)	30	22	BUCY	4-1BB	46 (3990)	CRS (24) NT (5)
Zhao (2020) **(** 1 **)**	China	122	HSCT: 26 (365) Non-HSCT: 27 (965)	122 (100.0)	107 (FCM)	55	40	Cy/Flu	4-1BB	67(34345)	CRS (113) Neutropenia (91) Sepsis (3) Pneumonia (3)
Zhang (2020) **(** 24 **)**	China	110	NR (261)	102 (92.7)	96 (FCM)	75	69	Cy/Flu	CD28/4-1BB	63 (36120)	CRS (102) NT (23) Seizure (13)
Wang (2020) **(** 10 **)**	China	23	42 (1067)	19 (82.6)	18 (FCM)	4	NR	FA	4-1BB	(2884)	CRS (23) Leucopenia and neutropenia (17) Anemia (7) Thrombocytopenia (9) NT (3)

allo, allogeneic; auto, autologous; BCA, B-cell aplasia; BM, bone marrow; CNSL, central nervous system leukemia; CR, complete remission; CRi, CR with incomplete count recovery; Cy, cyclophosphamide; CE, cyclophosphamide/etoposide; CRS, cytokine release syndrome; BUCY, cytosine arabinoside busulfan cyclophosphamide methyl-N-2-chloroethyl-N-cyclohexyl-N-nitrosourea; Vp16, etoposide; FA, fludarabine and cytarabine; HDflu, high-dose fludarabine; LDflu, low-dose fludarabine; MRD, minimal residual disease; NT, neurotoxicity; NR, not report; TL, testicular leukemia; N, number of patients.

All 14 studies enrolled for safety analysis were non-randomized, and their basic information is listed in **Table 2**. A total of 328 patients were bridged to HSCT in the 14 studies. Of the 10 studies reporting the median time between CAR-T therapy and HSCT, the interval time was within 3 months. All 14 studies reported TRM with an incidence rate ranging from 0 to 50%. The specific causes of TRM were reported in five studies and included GVHD, infections, veno-occlusive disease (VOD),and lung diseases. The most common causes were GVHD (n = 6) and infections (n = 5). Adverse events related to HSCT included aGVHD, cGVHD, infections, and VOD, etc. Five studies mentioned aGVHD, with an incidence rate ranging from 23.3 to 73.7%; of which the incidence rate of severe aGVHD (grade III/IV) ranged from 0 to 21.1%. Six studies mentioned cGVHD with an incidence rate from 9.5 to 88.9%. In total, five studies reported pathogens of infections, including bacteria, fungi, and viruses. Among them, 36 cases of cytomegalovirus (CMV) infection and 32 cases of EpsteinBarr virus (EBV) infection were reported.

**Table 2:** Characteristics of the included studies with safety outcomes.

Study	Number of transplant patients	Donors (N)	Conditioning regiments (N)	Median follow-up time(range)	Median days post-CAR-T to transplantation(range)	Transplant-related mortality(%)	Causes of death (N)	GVHD prophylaxis	aGVHD		N of cGVHD(%)	Infections(N)
									N (%)	N of Grade III-IV (%)	
Zhao (2020) **(** 1 **)**	55	Haplo (55)	MAC NMA	613 d (1001,403)	67 d (34345 d)	0 (0)		NR	35 (63.6)	4(7.3)	22(40)	Bacterial infections (8) Fungal infections (3) EBV infections (30) CMV infections (30)
Zhang (2020) **(** 24 **)**	75	Haplo (50) MSD (16) MUD (9)	MAC (age >5 years: TBI-based; age 5 years: Bu-based)	233.5 d (27478)	63 d (36120 d)	3 (4.0)	Septic shock (1) GVHD (2)	CsA, MTX, MMF	NR	NR	NR	NR
Gu (2020) **(** 6 **)**	30	Haplo (25) MUD (3) MRD (2)	BUCY	22 m (348)	46 d (3990 d)	5 (16.7)	NR	CsA, MMF, MTX (Haplo or MUD); CsA (MRD)	7 (23.3)	2 (6.7)	15 (50.0)	NR
Fabrizio (2020) **(** 17 **)**	15	RD (8) UD (7)	TBI-based (12) Chemo-only (3)*	39 m (148)**	57 d (30135 d)	3 (20.0)	VOD (2) GVHD (1)	CD34+selected TCD; CNI+MTX; CNI+MMF	5 (33.3)	2 (13.3)	4 (26.7)	EBV infection (1) CMV infections (4) Bacterial infections (7)
Ai (2020) **(** 15 **)**	9	Haplo (6) MSD (3)	TBI/Cy/Vp16 Bu/Cy+Vp16 TBI	262 d (150540)	32.5 d (2060 d)	0 (0)		CsA+MTX (MSD) MMF+CsA+MTX (Haplo)	2 (22.2)	0 (0)	8 (88.9)	Pulmonary infection (1) BSI (4) CMV+EBV infection (1)
Jiang (2019) **(** 8 **)**	21	Haplo (13) MSD (8)	Vp16+Bu+Cy	7.7 m (0.733.9)	44 d (3389 d)	2 (9.5)	cGVHD (1) Pulmonary infection (1)	Cy/Tacro,MTX (MSD) MMF, anti-CD25 monoclonal antibody, ATG (Haplo)	NR	0 (0)	1 (4.8)	Pulmonary infection (1)
Shadman (2018) **(** 22 **)**	19	MUD (9) UCT (5) MRD (3) mMURD (1) Haplo (1)	MAC (14) RIC (2) NMA (3)	36 m (NR)**	72 d (28138 d)	4 (21.1)	GVHD (1) Fungal infection (1) Sepsis (1) IPS (1)	CNI+MMF (5) CNI+MMF+sirolimus (1) CNI+MTX (9) CNI+MTX+abatacept (3) CNI+MMF+PtCy (1)	14 (73.7)	4 (21.1)	4 (21.1)	Viral and fungal Infections (11)
Pan (2017) **(** 20 **)**	27	Haplo (17) MUD (7) MSD (3)	TBI/Cy/Ara-C/MeCCNU/ ATG (24) Bu/Cy/Ara-C/MeCCNU/ ATG (3)	206 d (45427)	84 d (35293 d)	2 (7.4)	NR	NR	NR	NR	NR	NR
Park (2018) **(** 11 **)**	17	NR	NR	29 m (165)	74 d (44312 d)	6 (35.3)	TRM	NR	NR	NR	NR	NR
Cao (2018) **(** 16 **)**	2	MRD (2)	NR	244 d (105624)	65 d (30138 d)	1 (50.0)	GVHD and infections (1)	NR	NR	NR	NR	NR
Summers (2018) **(** 23 **)**	24	NR	NR	>1 y	NR	1 (4.1)	TRM	NR	NR	NR	NR	NR
Hu (2016) **(** 18 **)**	4	NR	NR	142 d (30181)	NR	0 (0)		NR	NR	NR	NR	NR
Qasim (2017) **(** 21 **)**	5	NR	NR	NR	NR (79 w)	1 (20.0)	TRM	NR	NR	NR	NR	NR
Zuo (2019) **(** 25 **)**	25	NR	NR	406 d (161,259)	NR (3197 d)	3 (12.0)	TRM	NR	NR	NR	NR	NR

Ara-C, Cytarabine; ATG, anti-thymocyte globulin; Bu, busulfan; BSI, blood stream infection; BUCY, cytosine arabinoside busulfan cyclophosphamide methyl-N-2-chloroethyl-N-cyclohexyl-N-nitrosourea; Chemo, chemotherapy; CMV, cytomegalovirus; CNI, calcineurin inhibitor; CsA, cyclosporin A; Cy, cyclophosphamide; D, day; DUCB, double umbilical cord blood; EMV, equine morbillivirus; Vp16, etoposide; Flu, fludarabine; Haplo, haploidentical donor; HUCT, haplo-umbilical cord transplant; IPS, idiopathic pneumonia syndrome; MAC, myeloablative conditioning; MeCCNU, Methyl-CCNU; MMF, mycophenolate mofetil; mMURD, mismatch unrelated donor; MRD, matched related donor; MSD, matched sibling donor; MTX, methotrexate; MUD, matched unrelated donor; NMA, nonmyeloablative conditioning; NR, no reported; PtCy, posttransplant Cy; RD, related donor; RIC, reduced-intensity conditioning; Tacro, tacrolimus; TBI, total body irradiation; TCD, T cell depleted; TRM, transplant-related mortality; UCT, umbilical cord transplant; UD, unrelated donor; VOD, veno-occlusive disease; W, week; Y, year.

*Clofarabine/melphalan/thiotepa and Flu/melphalan/thiotepa.

**Time from HSCT.

### Overall Survival

**Figure 2:**
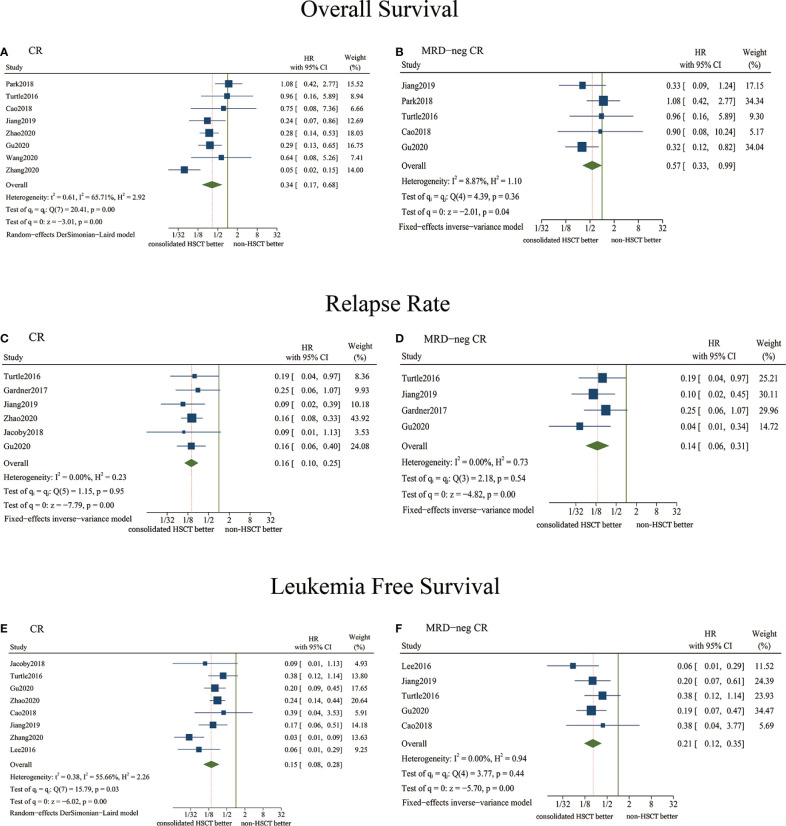
Forest plots of efficacy outcomes comparing consolidative HSCT and non-HSCT using pooled hazard ratios (HRs). **(A)** Overall survival (OS) analysis for patients achieving CR after CAR-T therapy; **(B)** OS analysis for patients achieving MRD-neg CR after CAR-T therapy; **(C)** Relapse rate analysis for patients achieving CR after CAR-T therapy; **(D)** Relapse rate analysis for patients achieving MRD-neg CR after CAR-T therapy; **(E)** Leukemia-free survival (LFS) analysis for patients achieving CR after CAR-T therapy; **(F)** LFS analysis for patients achieving MRD-neg CR after CAR-T therapy.

Eight studies reported data related to OS, in which patients achieved CR before receiving consolidative HSCT post-CAR-T therapy (**Figure 2A**). The pooled HR was 0.34 (95% CI, 0.170.68, P = 0.003), indicating a significantly better OS for patients who received consolidative HSCT. Based on the moderate heterogeneity (I^2^ = 65.71%, P = 0.005), the DerSimonian-Laird random-effects model was selected.

Five studies were enrolled for analysis when the inclusion criteria of the HSCT group only included patients achieving MRD-neg CR before consolidative HSCT therapy (**Figure 2B**). The pooled HR was 0.57 (95% CI, 0.330.99, P = 0.045), and there was low heterogeneity (I^2^ = 8.87%, P = 0.356). The pooled HR indicated a significantly better OS for patients who received HSCT after achieving MRD-neg CR.

### Relapse Rate

Six studies reported sufficient data regarding relapse in the two groups, in which patients received HSCT after achieving CR (**Figure 2C**). The pooled HR was 0.16 (95% CI, 0.100.25, P < 0.001) (I^2^ = 0.00%, P = 0.950). Thus, the patients who were bridged into HSCT had a significantly decreased likelihood of relapse.

Four studies were analyzed when the inclusion criteria of the HSCT group only included patients achieving MRD-neg CR before consolidative HSCT therapy (**Figure 2D**). The pooled HR was 0.14 (95% CI, 0.060.31, P < 0.001) (I^2^ = 0.00%, P = 0.535). The pooled HR indicated a significantly lower risk of relapse in the HSCT group after MRD-neg CR was achieved.

### Leukemia-Free Survival

Eight studies reported data related to LFS, in which patients achieved CR before receiving consolidative HSCT (**Figure 2E**). The pooled HR was 0.15 (95% CI, 0.080.28, P < 0.001). Based on the moderate heterogeneity (I^2^ = 55.66%, P = 0.027), the DerSimonian-Laird random-effects model was chosen. The pooled HR showed that the LFS results of the HSCT group were significantly better than those of the non-HSCT group.

Five studies were enrolled when the inclusion criteria of the HSCT group only included patients who achieved MRD-neg CR before consolidative HSCT therapy (**Figure 2F**). The pooled HR was 0.21 (95% CI, 0.120.35, P < 0.001), and there was low heterogeneity (I^2^ = 0.00%, P = 0.438). The pooled HR indicated significantly better LFS in the HSCT group after achieving MRD-neg CR.

### Analysis of the Costimulatory Domain of CAR-T Cells

OS was analyzed in six studies using the 4-1BB costimulatory domain of CAR-T cells (**Figure S1A** **)**. The pooled HR for the 4-1BB cases was 0.32 (95% CI, 0.210.49, P < 0.001). The relapse rate was analyzed by five studies based on the 4-1BB costimulatory domain of CAR-T cells (**Figure S1B**). The pooled HR for 4-1BB cases was 0.16 (95% CI, 0.100.26, P < 0.001). LFS was analyzed by five studies based on the 4-1BB costimulatory domain of CAR-T cells (**Figure S1C** **)**. The results revealed a pooled HR of 0.24 (95% CI, 0.16-0.35, P < 0.001) for 4-1BB cases.

The results confirmed the significant efficacy of consolidative HSCT in 4-1BB cases, such that the treatment prolonged OS, reduced the relapse rate, and increased LFS, while the efficacy of this treatment in CD28 cases remained unclear because of the scarcity of studies. Thus, we could not perform subgroup analysis of 4-1BB and CD28.

### Transplant-Related Mortality

**Figure 3:**
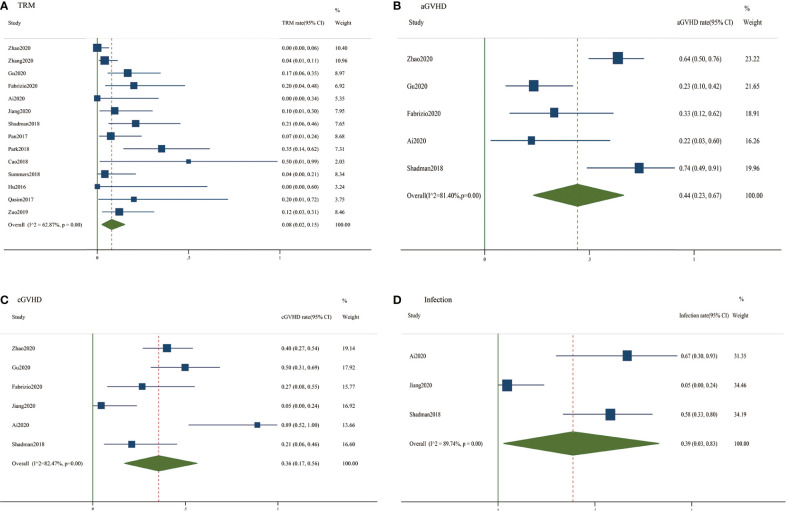
Forest plots of safety outcomes of consolidative HSCT using pooled incidence rates. **(A)** Pooled transplant-related mortality rates; **(B)** Pooled acute graft-*versus*-host disease rates; **(C)** Pooled chronic graft-*versus*-host disease rates; **(D)** Pooled infection rates.

Fourteen studies reported data concerning transplant-related mortality (**Figure 3A**). The pooled HR of the incidence rate of TRM was 0.08 (95% CI, 0.020.15). The DerSimonian-Laird random-effects model was selected based on the significant heterogeneity (I^2^ = 62.87%, P < 0.01).

### Acute GVHD

Five studies reported data concerning acute GVHD (**Figure 3B**). The pooled incidence rate of acute GVHD was 0.44 (95% CI, 0.230.67). Based on the significant heterogeneity (I^2^ = 81.40%, P< 0.01), the DerSimonian-Laird random-effects model was selected.

### Chronic GVHD

Six studies assessed chronic GVHD and were enrolled in this analysis (**Figure 3C**). The pooled incidence rate of chronic GVHD was 0.36 (95% CI, 0.170.56). The DerSimonian-Laird random-effects model was selected based on the significant heterogeneity (I^2^ = 82.47%, P < 0.01).

### Infections

Only three studies reported data related to infections after bridging to HSCT (**Figure 3D**). The pooled incidence rate of infections was 0.39 (95% CI, 0.030.83). Because of the significant heterogeneity (I^2^ = 89.74%, P < 0.01), the DerSimonian-Laird random-effects model was selected.

### Study Quality and Risk of Bias

The 11 cohort studies were evaluated using the Newcastle-Ottawa Scale (**Table S1**) and were all categorized as low risk. The eight single-arm studies were evaluated using the modified Institute of Health Economics (IHE) risk of bias tool and were also categorized as low risk (**Figure S2**).

We performed publication bias analyses using funnel plots, Eggers test, and Beggs test. The scattered points of the funnel plots were symmetrically distributed. The P values of Eggers and Beggs tests for all outcomes were >0.05, suggesting no significant publication bias. Beggs plots are shown in **Figure S3**.

### Sensitivity Analysis

Using different models did not affect the efficacy outcomes but did affect the safety outcomes. No small-sample effect was observed for the efficacy analysis, and a potential small-sample effect was observed for the safety analysis.

To evaluate the stability of the analysis, the studies were excluded one by one according to the order of Newcastle-Ottawa Scale scores and IHE assessment outcomes. Sensitivity analysis proved the analysis of relapse stable. However, confounding factors caused instability in other outcome indicators. Two studies (11, 24) showed heterogeneity in OS, one study showed heterogeneity in LFS (24), two studies showed heterogeneity in TRM (1, 11), one study showed heterogeneity in aGVHD (6), and two studies showed heterogeneity in cGVHD (8, 15). Regarding infections, excluding any study would alter the result because only three studies were included in the analysis.

## Discussion

To our knowledge, this is the first study to systematically analyze the efficacy and safety of consolidative HSCT after CD19 CAR-T therapy for patients with R/R B-ALL. Our findings suggest that bridging to HSCT after CAR-T therapy is an efficient and relatively safe method that prolongs OS and LFS and reduces the relapse risk without obviously increasing the risks of adverse events.

Achieving molecular remission is considered an ideal outcome for ALL patients and is superior to hematologic remission. Our study indicates that patients who received consolidative HSCT after achieving CR or MRD-neg CR had a longer OS and LFS and a lower risk of relapse. Because no adequate MRD grouping information was available and the sample data of MRD-positive CR patients were too small, it was difficult to compare the results of MRD-neg CR patients and MRD-positive CR patients by subgroup analysis directly. Some studies suggest that, after the administration of CAR-T cells, patients achieving MRD-neg CR might have a longer OS and LFS, while patients achieving MRD-positive CR had a higher chance of relapse (11, 27). Additionally, the state of MRD-neg CR could greatly benefit the outcomes of HSCT (26, 28, 29). In the enrolled studies, Zhao etal. (1) reported a longer OS and LFS of MRD-neg CR patients than those of MRD-positive CR patients, and Gu etal. (6) drew a similar conclusion. Thus, a patients state of MRD-neg CR is a strong indicator of a better prognosis after HSCT. However, this finding may not indicate that MRD-positive patients are unsuitable for HSCT. Because the risk of relapse is higher in these patients, they may require consolidative HSCT to improve the prognosis (30). Studies with larger sample sizes are needed to verify the outcomes of MRD-positive patients. Notably, qPCR may be more sensitive than FCM in MRD detection (5); thus, the detection method must be standardized.

Previous studies have shown that CD28 CAR-T cells can lead to robust expansion initially but short persistence and high relapse rates. By contrast, 4-1BB CAR-T cells have a prolonged duration *in vivo* and antitumor effects. Thus, we analyzed the 4-1BB costimulatory domain and further confirmed the prolonged OS and LFS, as well as lower risks of relapse for the 4-1BB group after HSCT. Because few studies meeting the inclusion criteria adopted CD28 as a costimulatory domain, we were unable to perform subgroup analysis of 4-1BB and CD28. In the future, more randomized studies are needed to determine the effect of the CAR costimulatory domain on the post-HSCT outcomes of patients.

Safety was a focus of this novel therapy. It was previously believed that CAR-T infusion and subsequent conditioning therapy might induce synergetic immune dysregulation and immunosuppression (31, 32), increasing the incidence of transplant-related complications. Severe CRS and long-term pancytopenia may also increase the risk of infection. Our study showed that the overall TRM rate was 8%, the aGVHD rate was 44%, the cGVHD rate was 36%, and the infection rate was 39%. The incidence rates of these complications were tolerable and not higher than those of patients receiving HSCT alone (33,37). In addition, Gu etal. (6) found that the 2-year treatment-related mortality did not differ significantly between the consolidative HSCT and non-HSCT groups [14.3% (95% CI, 7.621%) *vs.* 9.8% (95% CI, 3.216.4%); p = 0.804]. Furthermore, the reduction of TRM is a key issue and may benefit from the increasingly developed transplant techniques and maturity of preventive and curative measures for complications of HSCT and CAR-T.

Presently, the application of HSCT in the treatment of hematological malignancies is relatively mature, and there are no considerable technical obstacles at most transplant institutions. As a combinatorial regimen, maximizing the synergistic effects of CAR-T therapy and HSCT is important and is influenced by factors such as the patient status, donor sources, and transplantation timing, etc. The associated data in our review cannot be separated to perform subgroup analysis and draw quantitative conclusions. We will discuss those important factors based on trials enrolled in our study to optimize therapeutic regimens.

Regarding the patient status, age and pretransplantation history can influence choices concerning whether to undergo HSCT. Although we cannot propose a specific age limit, younger patients may benefit more from consolidative HSCT than elderly patients. Zhao etal. (1) proposed that age is an independent prognostic factor and that patients younger than 40 years had a significantly better prognosis (LFS, HR= 4.706, 95% CI, 1.630113.586; P = 0.004). Jiang etal. (8) reported an age older than 70 years as an exclusion criterion for HSCT. Regarding the pretransplantation status, receiving two or more transplantations may lead to a high relapse rate and incidence of complications after HSCT (38), and a pretransplant history before CAR-T cell infusion can also dampen the efficacy of a second transplantation after CAR-T cell infusion (26). Many patients with a transplant history have been excluded from many trials included in our study (1, 6, 8, 39). More studies are needed to determine whether patients with a history of transplantation can benefit from consolidative HSCT after CAR-T therapy. Other exclusion criteria included infections or complications, relapse from blinatumomab (40, 41), heavy tumor burden before CAR-T treatment (11, 42) and other contraindications (7, 8).

Regarding donor choices, in addition to HLA-identical donors, unrelated matched donors and haploidentical donors are gradually being accepted. In particular, for haplo-HSCT, almost all patients can obtain stable donor sources in a short time. The efficacy of haplo-HSCT is similar to that of HLA-matched HSCT (35); in our included trials, haplo-HSCT is the most common choice (**Table 2**). The graft can also be further optimized to improve the safety of transplantation. Using CD34 selected T-cell deletion (TCD) may be a feasible solution. Previously, it was believed that the depletion of CD34-positive T cells could result in slow immune recovery and a high infection rate (43). However, in the study of Fabrizio etal. (17), CD34-selected TCD allo-HSCT significantly reduced TRM without affecting disease control ability. If transplant-related risks are high and allo-HSCT is inappropriate, auto-HSCT may also be a strategy (16).

The timing of transplantation should also be considered because the optimal timing of HSCT after CAR-T infusion may greatly influence the outcome and safety of this therapy. Shadman etal. (22) found that a longer bridging time may deteriorate patient prognosis. Those bridging to allo-HSCT 80 days were associated with a higher risk of death (HR= 4.01, 95% CI, 1.1414.0; P = 0.03) and higher non-relapse mortality (HR= 4.4, 95% CI, 0.5421.1; P = 0.19) than those transplanted within 80 days, consistent with the study at another center (17). Over time, CAR-T cells may gradually fail, and the risk of relapse will increase accordingly (20). Therefore, after patients stabilize from CAR-T therapy, they should bridge to HSCT as soon as possible to continuously suppress tumor cells and improve the long-term prognosis of patients.

Our meta-analysis has some limitations. First, because few randomized controlled trials exist for CAR-T therapy due to its novelty, some bias may have been introduced because of the nature of our study. Patients in the HSCT group may achieve better outcomes, partly because fitter patients were more likely to be chosen for transplant. Measures were taken to reduce such bias. For example, we compared the HSCT *versus* non-HSCT groups based on both achieving CR or MRD-neg CR, a strategy that should reduce bias to a certain extent. Second, the limited number of included studies and small sample sizes of several studies may compromise the accuracy of the results, also resulting in an unclear conclusion of the CD28 subgroup. Third, the analysis was not sufficiently thorough because of incomplete information, including the age, pretransplantation history, donor, timing, and conditioning therapy of each group. Thus, we cannot provide detailed recommendations for consolidative HSCT. Despite these limitations, our study still provides guidance for clinical practice and directions for future research.

## Conclusion

Our study suggests that CD19 CAR-T cell therapy is an effective and safe bridge to HSCT. HSCT after CAR-T therapy can prolong OS and LFS and reduce the risk of relapse. MRD-neg CR after CAR-T cell therapy is a good prognostic indicator for HSCT. The incidence rates for adverse events did not increase significantly, and the safety of the combination therapy was acceptable. More randomized clinical trials with longer follow-up durations are needed to confirm these findings.

## Data Availability Statement

The original contributions presented in the study are included in the article/**Supplementary Material**. Further inquiries can be directed to the corresponding authors.

## Author Contributions

XXu, YL, and ST contributed to the study concept and design. SC, ZZ, ZH, XXiao, SH, and XXu contributed to the literature search, data collection, and data analysis. XXu, SC, ZZ, XXiao, and SH contributed to the drafting and review of the final manuscript. All authors contributed to the article and approved the submitted version.

## Funding

This research was supported by grants from the Clinical Research Startup Program of Southern Medical University by High-level University Construction Funding of Guangdong Provincial Department of Education (No. LC2016ZD027), Guangdong Science and Technology Department (No. 2017A020215183), the Major Program for Health Medical Collaborative Innovation of Guangzhou (No. 201704020216), Natural Science Foundation of Guangdong Province, China (No. 2018B030311042), Frontier Research Program of Guangzhou Regenerative Medicine and Health Guangdong Laboratory (No. 2018GZR110105014), National Students' Platform for Innovation and Entrepreneurship Training Program of China (202012121025) and Special Funds for the Cultivation of Guangdong College Students Scientific and Technological Innovation (No. pdjh2021b0101).

## Conflict of Interest

The authors declare that the research was conducted in the absence of any commercial or financial relationships that could be construed as a potential conflict of interest.

## Supplementary Material

Supplementary Table 1 NOS assessment outcomes of the 11 cohort studies.

Supplementary Figure 1 Forest plots of efficacy outcomes of consolidative HSCT after 4-1BB costimulatory CAR-T infusion. **(A)** Overall survival (OS) analysis; **(B)** Relapse rate analysis; **(C)** Leukemia-free survival (LFS) analysis.

Supplementary Figure 2 IHE assessment outcomes of the 8 single-arm studies.

Supplementary Figure 3 Beggs plots of all outcome indicators.
