# Supplementary material for: Consolidative Hematopoietic Stem Cell Transplantation After CD19 CAR-T Cell Therapy for Acute Lymphoblastic Leukemia: A Systematic Review and Meta-analysis
Source: Front Oncol. 2021 Apr 28;11:651944. doi: 10.3389/fonc.2021.651944 (PMC8139250; doi:10.3389/fonc.2021.651944)
Supplement: Supplementary Table 1 — NOS assessment outcomes of the 11 cohort studies. [file Table_1.pdf]

Author and Year	Selection	Comparability	Outcome	Total (maximum score 9*)
Park 2018 [11]	****	*	***	******** (8)
Lee 2016 [19]	****		***	******* (7)
Jacoby 2018 [7]	****		*	***** (5)
Turtle 2016 [9]	****	*	*	****** (6)
Gardner 2017 [5]	****		*	***** (5)
Cao 2018 [16]	****		*	***** (5)
Jiang 2019 [8]	****	*	*	****** (6)
Gu 2020 [6]	****	*	***	******** (8)
Zhao 2020 [1]	****	*	***	******** (8)
Zhang 2020 [24]	****	*	*	****** (6)
Wang 2020 [10]	****		*	***** (5)
