# Supplementary figures and images for: Consolidative Hematopoietic Stem Cell Transplantation After CD19 CAR-T Cell Therapy for Acute Lymphoblastic Leukemia: A Systematic Review and Meta-analysis
Source: Front Oncol. 2021 Apr 28;11:651944. doi: 10.3389/fonc.2021.651944 (PMC8139250; doi:10.3389/fonc.2021.651944)

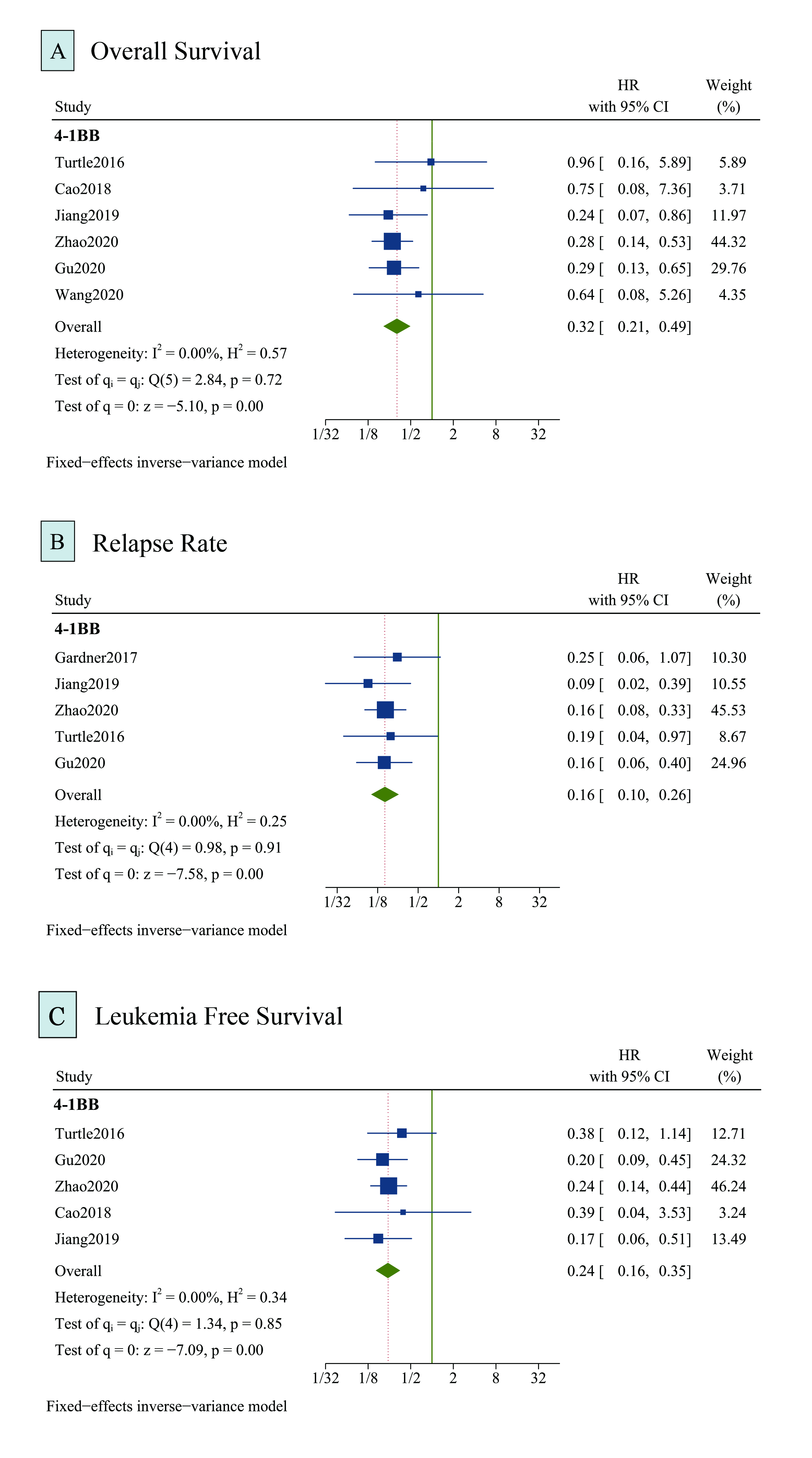

Supplement: Supplementary Figure 1 — Forest plots of efficacy outcomes of consolidative HSCT after 4-1BB costimulatory CAR-T infusion. (A) Overall survival (OS) analysis; (B) Relapse rate analysis; (C) Leukemia-free survival (LFS) analysis. [file Image_1.tif]

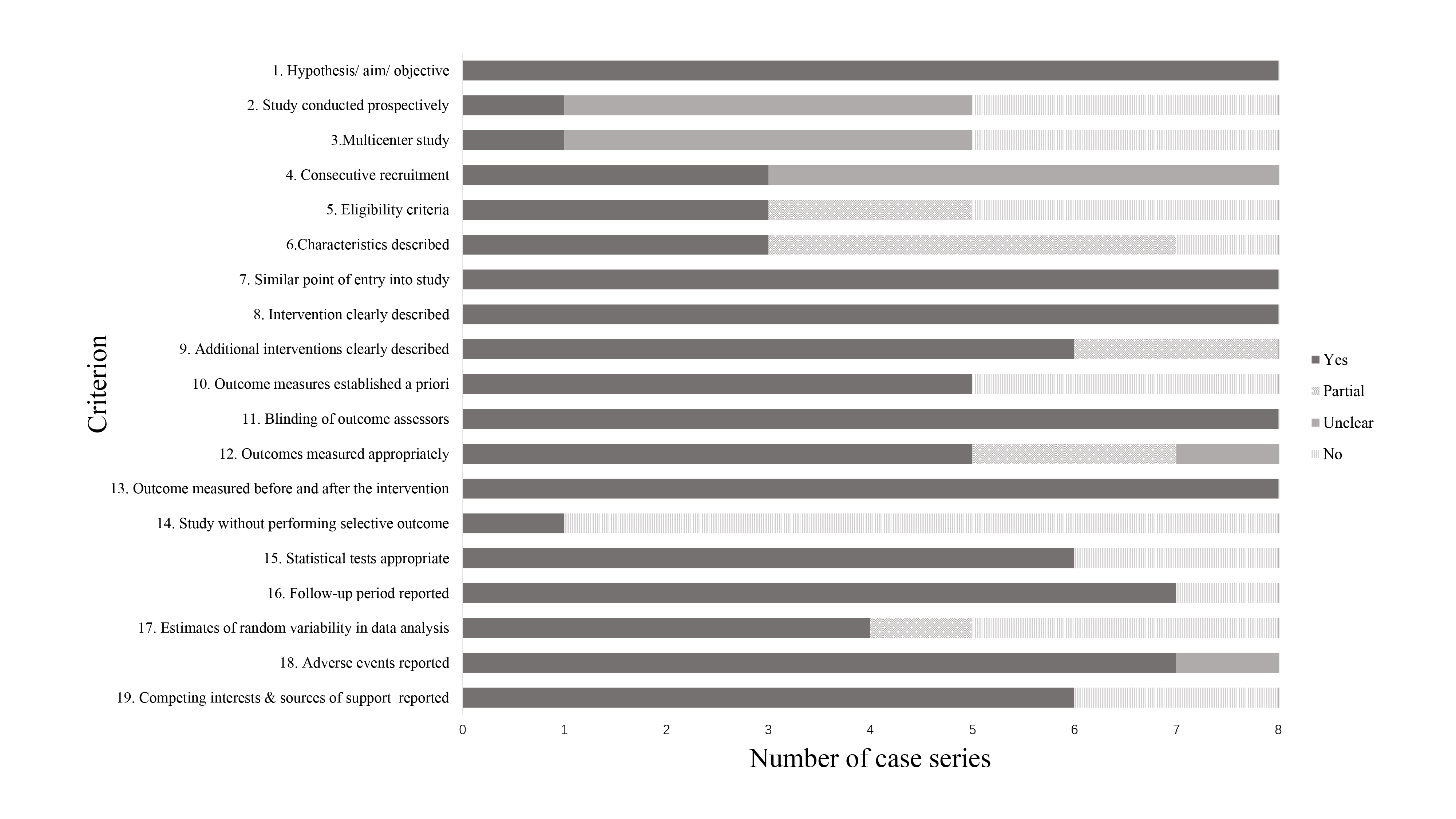

Supplement: Supplementary Figure 2 — IHE assessment outcomes of the 8 single-arm studies. [file Image_2.tif]

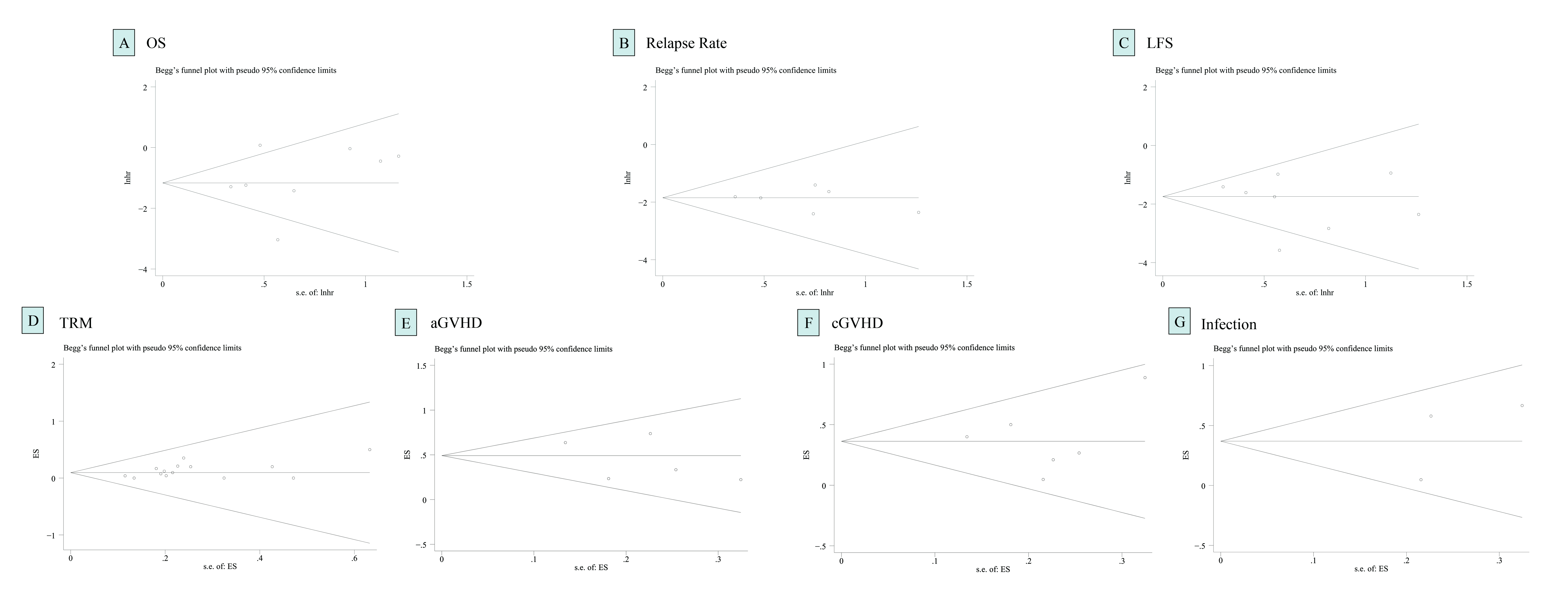

Supplement: Supplementary Figure 3 — Beggs plots of all outcome indicators. [file Image_3.tif]
